# Effects of aphid parasitism on host plant fitness in an aphid-host relationship

**DOI:** 10.1371/journal.pone.0202411

**Published:** 2018-08-23

**Authors:** Saori Watanabe, Yuuka Murakami, Eisuke Hasegawa

**Affiliations:** 1 Laboratory of Animal Ecology, Department of Ecology and Systematics, Graduate School of Agriculture, Hokkaido University, Sapporo, Japan; 2 Graduate School of Medicine, Department of Neuropharmacology, Hokkaido University, Sapporo, Japan; Helmholtz Zentrum Munchen Deutsches Forschungszentrum fur Umwelt und Gesundheit, GERMANY

## Abstract

Aphids are serious agricultural insect pests which exploit the phloem sap of host plants and thus transmit pathogens to their hosts. However, the degree to which aphid parsitism affects the fitness of the host plants is not well understood. The aphid, *Macrosiphoniella yomogicola*, parasitizes the mugwort *Artemisia montana* in Japan. During summer most mugworts carry aphids, but most aphid colonies die out after the budding of *A*. *montana* inflorescences in late summer. A few aphid colonies survive to late autumn, at which point sexuparae appear to later lay overwintering eggs after copulation. The death of the aphid colonies seems to be caused by biochemical changes in the phloem sap in the host plant coincident with the budding of inflorescences. The surviving aphid colonies may suppress the budding of inflorescences to allow persistence of their genetic line into the following year. Our investigations demonstrate that aphid parasitism did not affect host plant growth, but that it did significantly decrease the number of inflorescences and the average weight of floral buds. Our results indicate that aphid parasitism has a strong negative effect on the fitness of host plants. The manner in which the aphids suppress floral budding in their hosts is worth examining from the perspective of the evolution of aphid-plant interactions.

## Introduction

Arthropod-plant interaction is an important issue in basic and applied ecology from several perspectives: (a) understanding evolutionary arms races [1–5], (b) improving the yields of agricultural products and (c) elucidating the evolution of host-parasite relationships. Aphids transmit pathogens to host plants [6] and are therefore recognized as serious agricultural pests [7, 8]. Thus, the manner in which host plants resist aphid parasitism and the ways in which aphid species overcome the resistance of their hosts are important issues for understanding evolutionary arms races [1, 2]. Because aphids exploit the phloem sap of their hosts they would be expected to negatively affect the fitness components of host plants. Although many examples show that parasites do negatively affect such fitness components [7–**9**], detailed quantitative data is required to understand the current complex interactions between parasitic aphids and their host plants.

The aphid, *Macrosiphoniella yomogicola*, parasitizes the mugwort, *Artemisia montana*, in cold regions of Japan (high-elevation areas in Honshu and lowlands in Hokkaido). In Hokkaido stem mothers of *M*. *yomogicola* hatch from overwintered eggs and produce offspring asexually on the mugwort hosts. From mid-June to early July, winged individuals are produced asexually and disperse to distant mugwort plants. During the summer, the aphid generations cycle by asexual reproduction and most mugworts are parasitized by aphid colonies. However, a previous study [**10**] and our preliminary observations over several years showed that aphid colonies decreased in individual numbers from late summer to autumn in the field. This change may be caused by a physical or biochemical change in the phloem sap of the host coincident with budding of inflorescences. Such biochemical changes associated with flowering have been reported in tobacco plants [**11**]. Only those aphid colonies which survive until mid-October can produce sexuparae, the females of which lay overwintering eggs after mating with males. Therefore, the aphid cannot reproduce unless it overcomes the resistance (the budding of inflorescences) of the host plant. Thus, it is predictable that there is an evolutionary arms race between the aphid and the mugwort.

Some insects, including aphids, have been known to manipulate the developmental system of their hosts to create galls within which the parasites can obtain phloem sap and be protected from predation [12,13]. *M*. *yomogicola* might manipulate the host plant to suppress the budding of inflorescences in ways similar to the above example in order to allow their genetic lines to continue into the next year.

In this study, we investigated the relationship between injury caused by aphids and fitness components of the host plant during a period just before sexual reproduction of the aphids. To investigate the effects of injury caused by aphids on the growth of the host plants, the number of inflorescences, and the numbers and weights of the floral buds were measured and compared between shoots with and without aphids. We will discuss the evolution of adaptations of both the host and the parasite related to this arms race.

## Materials and methods

Our experiments are suitable for publication following Hokkaido university's guidelines. The university permitted our use its property at the study field site.

### Back ground of the subject aphids

*M*. *yomogicola* is distributed in cold regions throughout Japan. In Hokkaido, this species parasitizes the mugwort *A*. *montana* and is very common in the lowlands. *M*. *yomogicola* displays color polymorphisms [10] and the greater the color diversity in a population, the slower is the rate of population decline in a season [10]. In Hokkaido, there are basically two color-morphs, red and green. *M*. *yomogicola* individuals cannot survive without the support provided by ant attendance [14]. The most common attending ant species, *Lasius japonicus*, strongly prefers aphid colonies with approximately 65% of the green morph, and thus both of morphs are maintained in attended populations [14]. An aphid stem mother hatches from an overwintered egg on a mugwort and asexually produces offspring which share her coloration. From mid-June to early July winged aphids are produced asexually and disperse to other mugwort plants. Thus, most mugworts are parasitized by mixed-color aphid colonies during the summer.

### Back ground of the host plant, *A*. *montana*

The host plant, *A*. *montana*, is a common perennial in the study area (a property belonging to Hokkaido University). Individuals produce new shoots from overwintered living roots in the spring (late April) and grow to a height of approximately 1m during summer when most of them are parasitized by *M*. *yomogicola*. In late summer (mid-August) the host plant begins to produce several inflorescences with many floral buds, and after producing seeds, the aboveground parts of the plants expire.

Following the budding of the mugwort inflorescences the number of aphids decreases rapidly and many aphid colonies die out by early autumn. A few survive to mid-October, and they produce sexuparae which lay overwintering eggs. The rapid decline in aphid colony numbers are likely due to changes in biochemical components of phloem sap in the host plant coincident with the budding of inflorescences [**10**]. The aphid cannot reproduce unless it survives this critical period. Thus, determining the cause of survival is important when considering the aphid’s adaptive strategy. However, if the budding of inflorescences is the cause of extinction of the aphid colonies, then *M*. *yomogicola* might have evolved mechanisims to suppress the budding, in order to survive. Thus, there is likely an arms race between the aphid and the mugwort.

### Data sampling

From 12 August to 12 October 2017, we investigated a total of 32 mugwort shoots that were infected by *M*. *yomogicola*. The start day (12 August) is the day at which the first budding of inflorescences was observed in the study area. Within the ca. 1.8km^2^ study area two mugwort-communities were used, and the infected shoots were selected randomly from among those with relatively large numbers of aphids (ca. more than 50). We then counted aphids on each shoot with 3 to 4 days interval until 1 September 2016.

All of the shoots on which aphid colonies were present on 12 October 2016 were investigated (12 and 5 in Site 1 and Site 2, respectively). For those shoots without aphids, we selected 24 and 11 shoots in Site 1 and Site 2 respectively, with spacing of approximately 1m to reduce the possibility of double counting from the same genetic clone. This method does not guarantee that the all shoots were from separate clones, but a recent study has shown that genetic clone individuals showed a large degree of phenotypic variation [15]. Thus, we treated each shoot as an independent statistical datum point.

We selected each shoot randomly from a narrow area (ca. 30cm^2^) of each point. At Site 1, the mugwort shoots were found within a narrow area (ca. 1m x 3m) and we selected them randomly within the community. This sampling strategy was adopted because in our experience almost all aphid colonies comprising only a few individuals (ca. less than 30) would become extinct by the time of emergence of sexuparae in mid October. During the study period we recorded the numbers of aphids (ca. 2 to 3mm in length) on each mugwort in the field from 9 to 15 September 2016.

Using these data, we calculated the survival rates of the aphid population. When no aphid was found on a shoot, we considered that the subject colony had become extinct. Because several parasitized mugworts were added to our samples over the course of the investigation, we used the survival rate rather than the absolute numbers of aphids, since we wished to determine how many colonies had died out during the observation period.

Next, we compared several characteristics of those host plants with and without *M*. *yomogicola* during the production period of sexuparae. From 9 to 15 October 2017 we measured host plant characteristics at two sites on the Hokkaido University Campus (the backyard of the University Museum (Site 1) and the proximity of the Faculty of Engineering building (Site 2)). Eleven and 5 mugwort shoots (all the infected shoots at each site) with aphid colonies and 24 and 11 shoots without aphid colonies were measured in sites 1 and 2, respectively. The sampling numbers were determined to enable us to make statistical tests between the two groups. We selected several characters reflecting growth, and determined a fitness component for each shoot, since the aphids exploit phloem sap of the host shoots, and thus may produce negative effects on these parameters. The measured characteristics were as follows: height of the plant (H; to the nearest cm by using a tape measure), stem width 5 cm from the ground (W; to the nearest 0.01 mm by use of a digital caliper (Digimatic caliper^TM^, Mitutoyo, Kanagawa, Japan), number of inflorescences within the top 5 cm of the plant (Inf), number of floral buds on these inflorescences (fb), and the total dry weight of buds (measured to the nearest 0.001 g by using a digital balance (UX220H, SHIMADZU, Tokyo, Japan) after 24 h of drying at 80°C in a drying oven (SK401, YAMATO, Tokyo. Japan)). We then, calculated H/W (HW) as an index of plant condition.

### Analyses

The survival rates of aphid colonies were regressed on the days since the start of the investigation, and the significance of the slope of the linear regression was tested statistically. To determine the factor which most strongly affected the number of inflorescences we conducted a multivariate generalized linear model (GLM) analysis, based only on those mugworts without aphids, using a normal distribution. We set Inf as the dependent variable and H, W and their interaction term as the independent variables. The best model was determined by comparing Akaike’s information criterion (AIC; [16]) for each model. The model with the lowest AIC was selected as the best. We then regressed the number of inflorescences on the best estimator (detected by the above process) of Inf separately for each site. Differences in the slopes and the intercepts of the two regression lines were compared by ANCOVA, to determine whether or not the data could be combined. The measured characters were then compared between those mugworts with and without aphids.

Finally, we compared a fitness component characteristic (the total weight of floral buds) of those mugwort shoots with and without aphids. This fitness index reflects both the number and the weight of floral buds.

Welch’s *t*-test (unequal variances *t*-test) was used to compare H, W, HW and the average fb weight between mugwort’s shoots with and without aphids. As the Inf are not distributed normally at both the sites (Shapiro-Wilk test; for Site 1, W = 0.8221, p = 0.0184: for Site 2, W = 0.8852, p = 0.0106) the differences in the means of Inf were tested using the Mann-Whitney U-test. All of the statistical analyses were conducted by using R (ver. 3.2.1; [17]).

## Results

**Fig 1:**
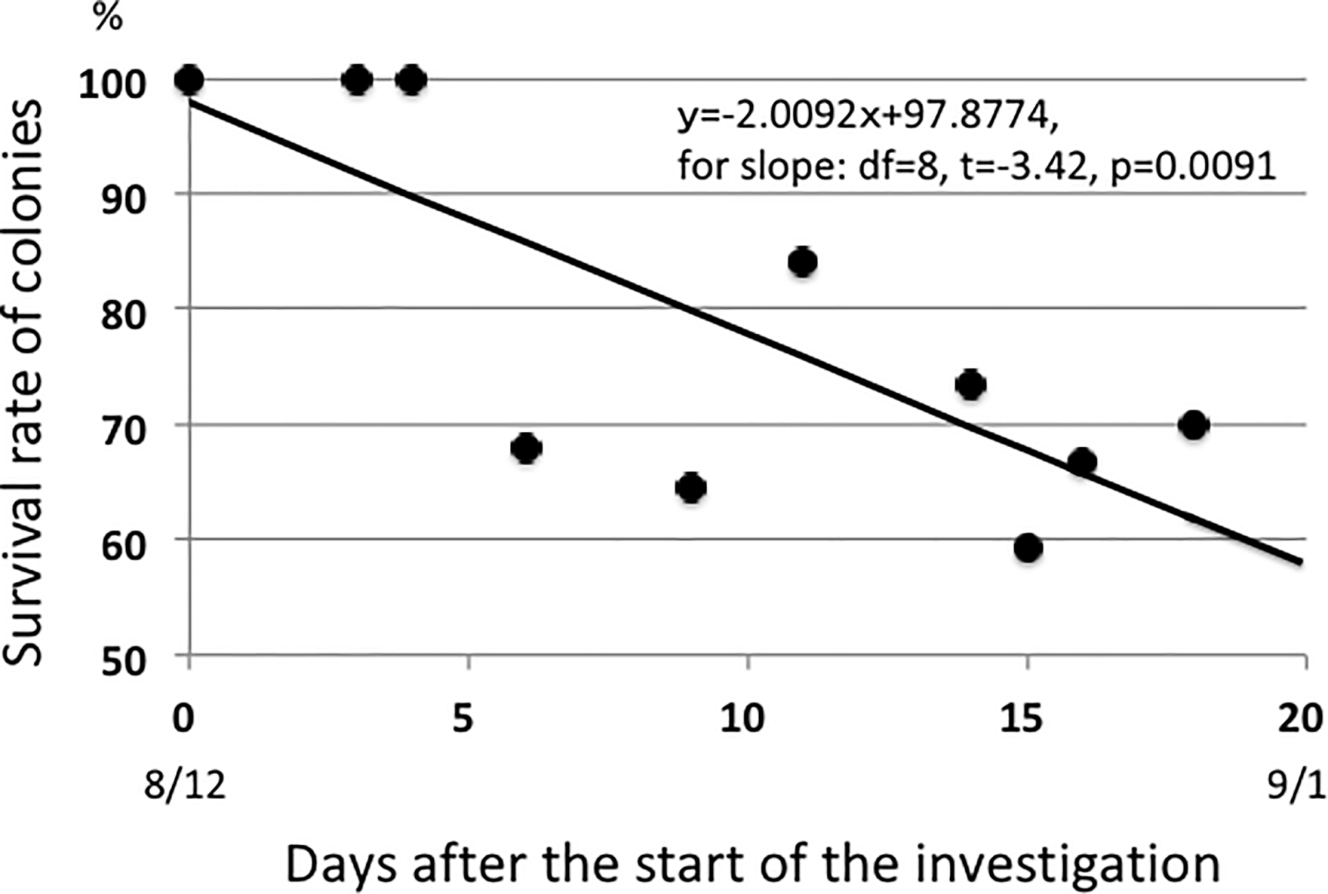
A pattern of decreasing numbers of *M*. *yomogicola* colonies on the host mugwort shoots in Autumn 2016. The numbers of parasitic colonies rapidly decreased with the budding of inflorescences of the host plant (the first budding was confirmed on 12 August). During the investigated period, the survival rate occasionally increased. Although this may appear strange, a colony that has died out is frequently re-parasitized by a few aphids before the next observation day, likely due to the movement of aphids from other colonies or to the transport of aphids from other colonies by attending ants.

Fig 1 indicates a significant negative relationship between survival rate of aphids and time (Rate = -2.0092×Days+97.877; for the slope, df = 8, t = -3.42, p = 0.0091). After the first budding of the host inflorescences (12 August) many of the aphid colonies died out rapidly (Fig 1). The GLM analysis indicated that the model with only HW as a dependent variable is the best model to estimate the number of inflorescences (see S1 Table).

**Fig 2:**
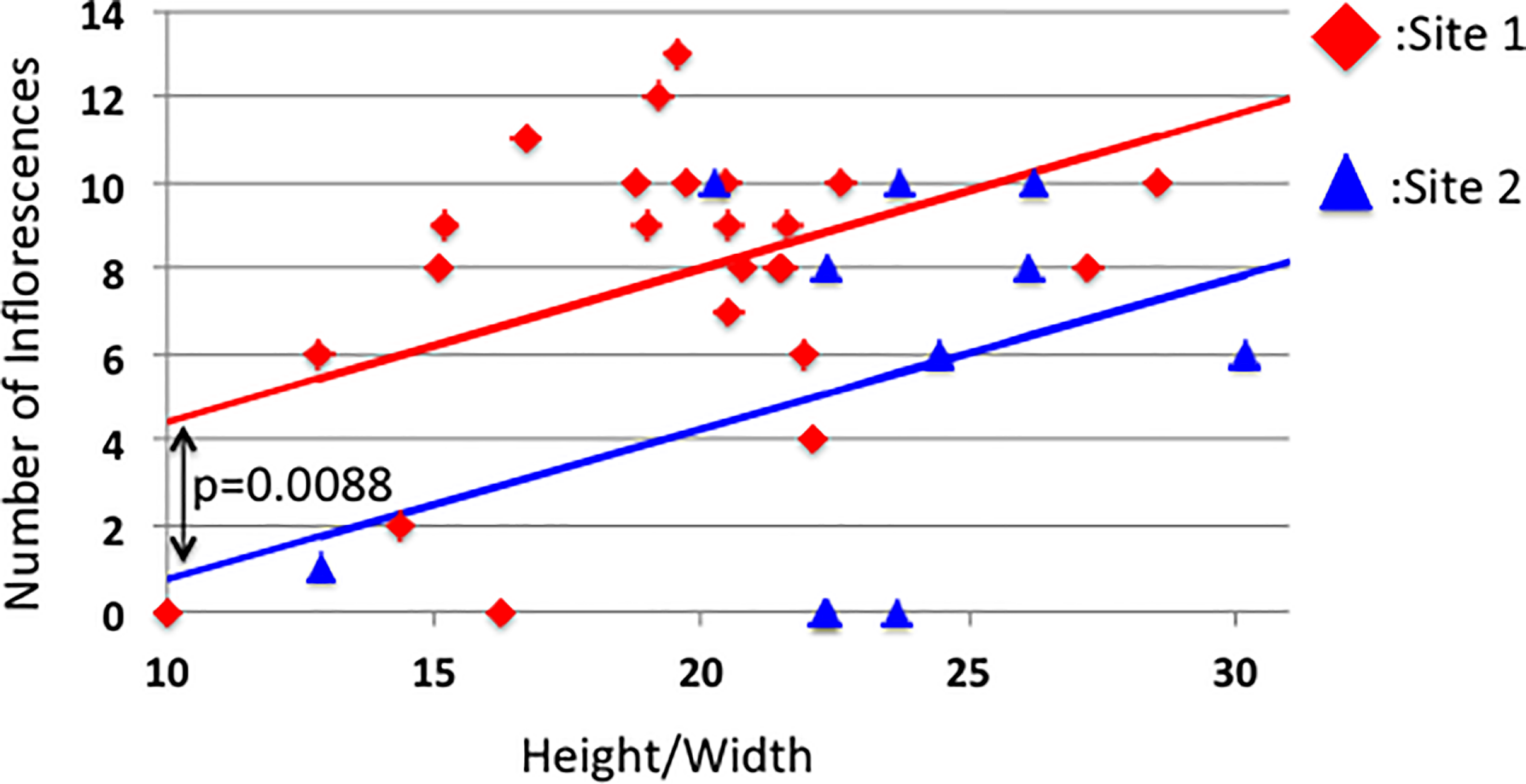
The regression lines for the numbers of inflorescences within the top 5 cm of each shoot on the height/basal width of each shoot. The slopes of the two study sites are not significantly different (for Site 1: 0.3602; for Site 2: 0.3522; ANCOVA: F = 0.0007, df = 44, p = 0.9795), but the intercepts are significantly different (for Site 1: 0.7996; for Site 2: -2.7839; df = 44, t = -2.789, p = 0.0088). These results mean that the number of inflorescences increases as the H/W ratio increases in the same ratio but the expected number of inflorescences to a HW value are different between the sites. Thus, we cannot combine the data.

We compared regressions of number of inflorescences on H/W ratios for both the sites separately. Fig 2 presents two regression lines of HW on Inf, one for each study site. The regression slope of HW on Inf is significantly positive in a generalized linear mixed model by setting the site as a random effect with poisson distribution with log-link function (the slope = 0.0486, S.E. = 0.0161, z = 3.029, p = 0.0027). Although the slopes of the two lines are not significantly different (for Site 1: 0.3602; for Site 2: 0.3522; F = 0.0007, df = 44, p = 0.9795), the intercepts are significantly different between the sites (for Site 1: 0.7996; for Site 2: -2.7839; df = 44, t = -2.789, p = 0.0088). These results show that the number of inflorescences increases as the H/W ratio increases but the difference between the intercepts means that we cannot combine the data across the sites to analyze the relationship between HW and Inf. Therefore, we analyzed data from the two sites separately. The difference in the intercept would be caused by a difference in nutrients in soils between them because Site 1 is a grass field at a roadside at where soil nutrients will return from fallen leaves in autumn, but in Site2 the mugworts had grown from the cracks in an asphalted road side where little nutrient feedback from fallen leaves would be expected.

**Fig 3:**
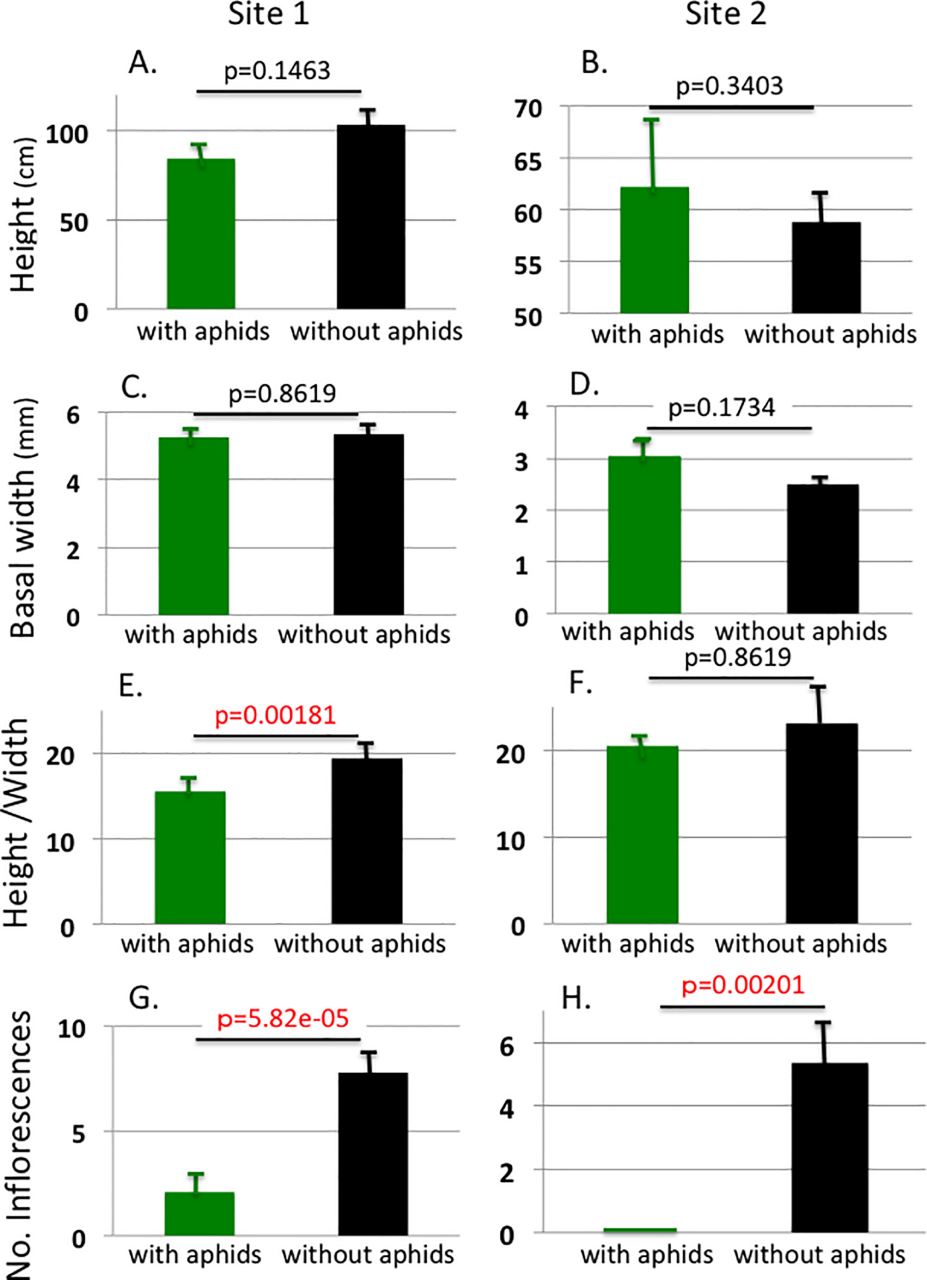
Comparisons of the measured characters between the shoots with aphids (green bars) and without aphids (black bars). The regression coefficients are significant for both the sites (Dite 1; slope = 0.3602, p = ). The whiskers represent the S.E. At both sites, the height and the basal width did not correlate with aphid parasitism (3A-D). However, at Site 1, height/width (an index of the physical condition of the host plant) was significantly affected by aphid parasitism (3E, F). At both sites, the number of inflorescences was highly significantly small on the shoots on which the aphids had remained to sexuparae productions (Fig 4G and 4H).

Fig 3A–3H present the means of the measured characters for the mugworts with and without aphids at both of the investigated sites. Aphid parasitism did not significantly affect H or W at either site, but did significantly correlated with HW at Site 1. The number of inflorescences is significantly different between the mugworts with and without aphids (p = 0.000059 for Site1 and P = 0.002 for Site2). However, as there is a significant difference in HW at Site 1, these differences might be due to this HW difference. Thus, we calculated the residuals of Inf values from the regression of Inf on HW (the lines in Fig 2). The mean residuals were then compared between the mugworts with and without aphids for both of the sites. Note that we used the regression lines calculated for only the mugworts without aphids because we intended to understand the degree to which the aphid parasitism negatively affects the plants’ fitness. Thus, the regression of uninfected mugworts on Inf should be used as a reference.

**Fig 4:**
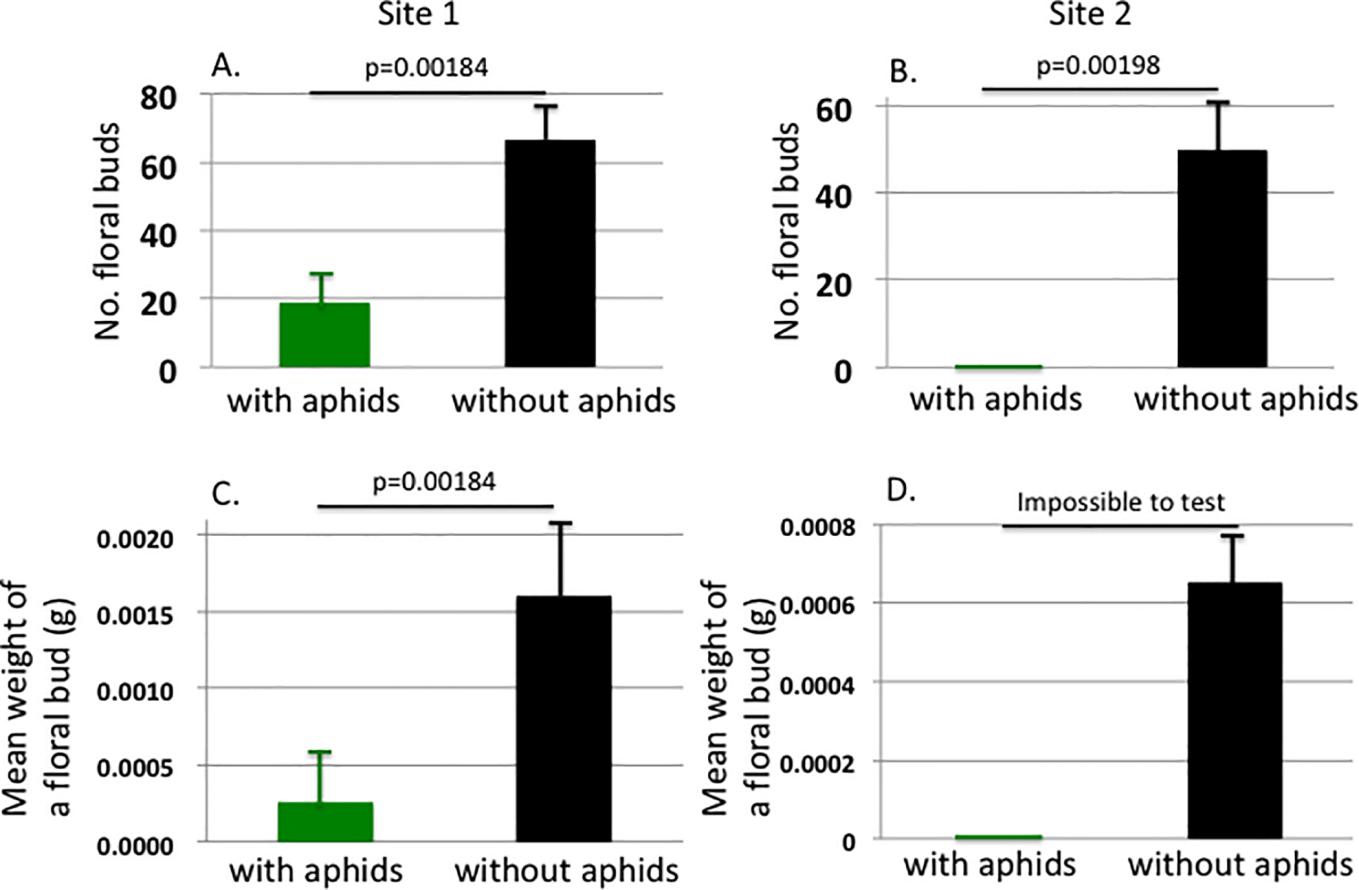
Comparisons of the floral characters between the shoots with aphids (green bars) and without aphids (black bars). At both the sites, the number of floral buds and the mean weight of a floral bud were negatively affected by injury by *M*. *yomogicola* (3A-D). For Site 2, we could not test the significance of the effect on the mean weight of a floral bud because none of the parasitized shoots had any buds or inflorescences (3D).

We compared the number and mean weight of floral buds between the shoots wit and without the aphids. Fig 4A and 4B show the effects of aphid parasitism on the number and mean weight of floral buds. Both the number and the mean weight were affected negatively by the presence of aphids. The mean weight at Site 2 was impossible to test, because in this site, none of the parasitized shoots had budded any inflorescences; thus, weight data for floral buds could not be obtained for the shoots with aphids at this site.

**Fig 5:**
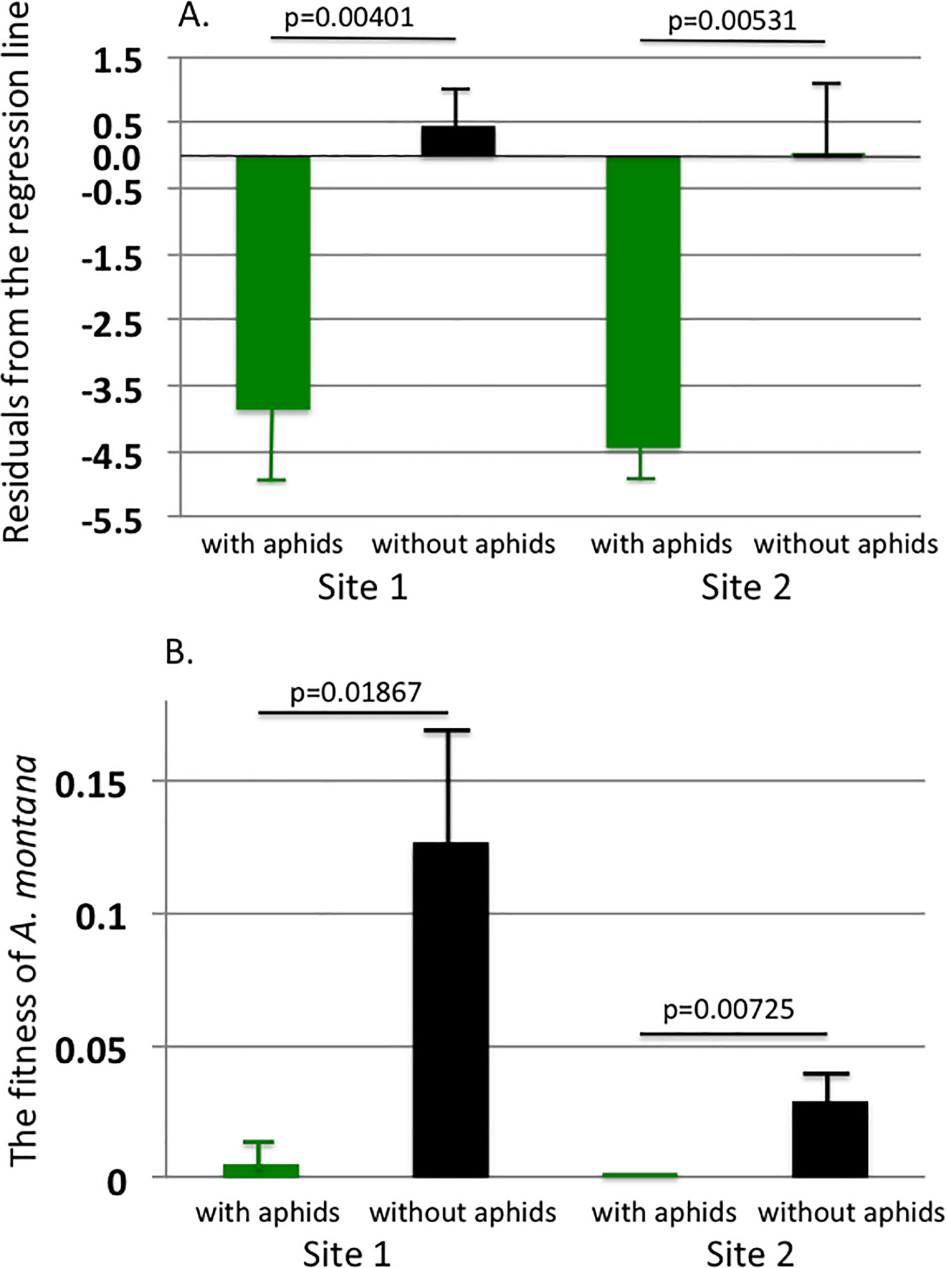
Comparisons of fitness of *A*. *montana* between the shoots with aphid prasitism (green bars) and without aphids (black bars). Fig 5A shows comparisons of the mean residuals of the no. of inflorescences of the shoots with and without the aphids. The residual values were calculated from the regression line in Fig 2 (the regression of the number of inflorescences on HW among the shoots without the aphids). The residuals of the parasitized shoots were calculated for these lines because we intended to determine the effect of aphid parasitism compared to the normal shoots. Fig 5B shows the fitness component (total weight of floral buds) between shoots with and without aphids at each site. The shoots with aphids almost completely lost their fitness.

We calculated residuals of no. of inflorescences on HW for the uninfected shoots without the aphids for both the shoots with and without the aphids to compare an effect of the aphid presence on inflorescence buddings. Fig 5A shows the mean residuals of Inf at the two sites. The residuals were calculated from the regression of no. of inflorescences on HW for “the uninfected shoots”, and thus this values for the aphid infected shots show a degree of decrease in these fitness components when an uninfected shoot will continue to be parasitized by the aphids.

Fig 5B shows a comparison of the mean observed fitness components of the mugworts with and without aphids. Again, at both sites, the fitness components of the shoots greatly reduced by the aphid parasitism. For both the sites, the mean residuals are negative for the shoots with aphids, and the residuals are significantly different between the parasitized and the uninfected shoots. Therefore, the aphid presence negatively affects the budding of inflorescences of the host shoot and their fitness component.

## Discussion

Our results demonstrate that aphid parasitism has a severe negative effect on the fitness of host mugwort shoots. Fitness (estimated as total dry weight of the floral buds) decreased to 26.73% and 0% at Site 1 and Site 2, respectively (Fig 4B). This was due to decreases in both the number of inflorescences and the average weight of floral buds (the latter was found in Site 1 only). Fig 4A shows that, compared to the shoots without aphids, the parasitizing aphids suppressed the budding of inflorescences of the host shoots. Although the average HW was significantly smaller in those shoots with aphids than in those without them, the results were statistically controlled for this difference by using a residual analysis. Thus, the aphids can control the hosts developmental system by some means of manipulation. Gall-making arthropods manipulate the developmental systems of host plants in order to induce the development of galls [12,13]. *M*. *yomogicola* might similarly manipulate *A*. *montana*, although there could be other possibilities, e.g., the aphids might remove photosynthate from the host shoots preventing growth of inflorescences. These hypotheses require verification to understand the host-aphid interactions in more detail.

The reason for this manipulation by the aphids seems to be a biochemical change in the phloem sap of the host plant associated with the budding of inflorescences. Fig 1 shows that after the budding of inflorescences, many colonies of *M*. *yomogicola* died out. Within a few weeks, the number of aphid colonies had decreased by 40%. This decrease continues after 1 September, and most of the aphid colonies had become extinct by mid October when sexuparae of *M*. *yomogicola* emerge. The decrease may relate to the onset of Autumn with shorter and colder days, but the survived colonies until sexuparae production decreased once in the aphid numbers but re-increased from a day point. Thus, seasonal changes could not explain the decrease of the aphid colonies because if the climate changes is the cause all of the aphid colonies should become extinct. In addition, if the aphid does not injure the host plant it might be subject to infection until sexuparae production. Probably some chemical substances included in the aphid saliva might control the developmental system of the host plant, as in gall-making aphids. However, that issue is beyond the scope of this study and will be examied elsewhere. Thus, an arms race expected between *A*. *montana and M*. *yomogicola* because a shoot will lose between 74.27 and 100% of its fitness components in a year when it allows the presence of the aphid colonies, but the aphids lose fitness completely when they become extinct before sexuparae production. These severe negative effects on the opponent’s fitness components would lead to an evolutionary arms race between the two parties [1, 2, 18].

*A*. *montana* is a perennial plant that forms a clonal plant community [19] in which the clonal shoots are connected to each other by roots. This feature of *A*. *montana* enables it to evolve traits via kin selection [20–24]. Self-sacrificing traits for an individual shoot can evolve if these sacrifices increase the fitness components of associated shoots in a clonal community. Kin-selected traits have been reported for plants [21–23]. For example, an eaten plant releases a chemical substance from the resultant injured part, increasing production of alkaloids in adjacent kin [24].

If injury by *M*. *yomogicola* provides some benefits to a clonal community of *A*. *montana*, then a clonal community may sacrifice some of the fitness components from a portion of the clonal shoots. Are there such presumable benefits to *A*. *montana* from injury by *M*. *yomogicola*? One possibility stems from the fact that *M*. *yomogicola* is an aphid with obligatory ant attendance [14]. Attending ants repel most of the predators of *M*. *yomogicola* [14]. Similarly, they may repel herbivorous insects that eat *A*. *montana* because such insects indirectly have negative effects on honeydew production by *M*. *yomogicola*. If a clonal community of *A*. *montana* gains fitness benefits from the attending ants on *M*. *yomogicola*, then sacrificing a subset of shoots in order to survive aphid parasitism might evolve through kin selection. Aphid-attending ants have been reported to protect host plants from herbivorous insects which are not predators of the aphids [25].

The evolution of a kin-selected trait can be explained by the well-known Hamilton rule (**br**-**c**>0; **b** = the fitness benefit of a recipient by altruistic traits of the donor, **c** = the fitness cost of a donor, **r** = genetic relatedness between the donor and the recipient) [20]. In the case of *A*. *montana*, **r** = 1 among clonal shoots. Thus, if **b** > **c**, then altruistic traits of a clonal shoot can evolve. We can examine this possibility by investigating the fitness cost of a clonal community (not of a shoot) and the benefit of *M*. *yomogicola* injury on a clonal community. We can estimate the latter by removing aphids by rubbing a sticky liquid at the base of shoots to remove attending ants from an aphid colony. Aphid colonies will become extinct using this method because aphid colonies die out due to predation soon after the removal of attendant ants [14]. The first part (cost of the aphid parasitism for a shoot) was estimated in this study. Note that the occurrence of *M*. *yomogicola* injury on most mugworts during summer could suggest the existence of benefits which allow injury during the growth period of *A*. *montana*. The host plant could benefit from the removal of aphids only after the start of the reproductive season (after the budding of inflorescences). In fact, the numbers of aphid colonies rapidly decreased during the reproductive season (Fig 1). However, it might be better for the host plant to sustain a portion of the aphid population in order to receive benefits from them in the following year, while sacrificing some fitness from the infected shoots. The results of such studies could provide new insights into plant-insect interactions. If the above is the case, then this aphid-plant relationship is a symbiosis rather than a one-sided relationship as with this ant-aphid symbiosis [26]. This interesting possibility awaits clarification through additional studies.

## Supporting information

S1 Table AIC values for each multivariate GLM. The best model is the one with the lowest AIC, has only HW as the best predictor of Inf.
(DOCX)
