# Supplementary material for: Effects of aphid parasitism on host plant fitness in an aphid-host relationship
Source: PLoS One. 2018 Aug 23;13(8):e0202411. doi: 10.1371/journal.pone.0202411 (PMC6107194; doi:10.1371/journal.pone.0202411)
Supplement: S1 Table — The best model is the one with the lowest AIC, has only HW as the best predictor of Inf. (DOCX) [file pone.0202411.s001.docx]

S1 Table. GLM models to predict no. of inflorescences (Inf) and those AIC values. The best model is the model with the lowest AIC (Inf = HW+Int.; represented by red characters).

| H | W | HW | H*W | H*HW | W*HW | H*W*HW | Int. | AIC |
| --- | --- | --- | --- | --- | --- | --- | --- | --- |
| 4.244e7 | -0.3752** | -8.118* | 0.3088* | 0.0739* | -4.244e7 | -0.01655* | 0.016** | 127.06 |
| 5.108e7 | -5.142 | 0.1012 | 6.637e3 | -0.010 | -5.108e7 | _ | 9.624 | 129.83 |
| 0.4115 | -5.6816 | -0.1242 | 0.0054 | -0.0089 | _ | _ | 13.384 | 129.33 |
| 3.191e7 | -1.809 | 0.0273 | 6.256e3 | _ | -3.191e7 | _ | 0.1032 | 132.63 |
| 5.009e7 | -4.389 | 0.1084 | _ | -9.98e-3 | -5.009e7 | _ | 6.022 | 127.98 |
| 0.0664 | -2.398 | -0.114 | 0.0055 | _ | _ | _ | 12.719 | 131.14 |
| 0.4377 | -5.0604 | -0.1148 | _ | -0.0089 | _ | _ | 10.394 | 127.43 |
| 3.102e7 | -1.104 | 0.0342 | _ | _ | -3.102e7 | _ | 6.921 | 130.74 |
| 0.0929 | -1.7658 | -0.1047 | _ | _ | _ | _ | 9.6815 | 129.22 |
| 0.0470 | -1.9723 | 0.0051 | _ | _ | _ | _ | 10.3916 | 129.19 |
| 0.04623 | _ | 0.5526 | _ | -0.0022 | _ | _ | -3.1753 | 129.78 |
| _ | -1.7658 | -0.1047 | _ | _ | 0.0929 | _ | 9.6815 | 129.22 |
| 0.0224 | _ | _ | _ | _ | _ | _ | 5.4744* | 130.04 |
| _ | -1.078 | _ | _ | _ | _ | _ | 8.369** | 131.41 |
| _ | _ | 0.360* | _ | _ | _ | _ | 0.7996 | 126.20 |
